# A Feasibility Study of Non-Invasive Continuous Estimation of Brachial Pressure Derived From Arterial and Venous Lines During Dialysis

**DOI:** 10.1109/JTEHM.2020.3035988

**Published:** 2020-11-04

**Authors:** Jill Stewart, Paul Stewart, Thomas Walker, Daniela Viramontes Horner, Bethany Lucas, Kelly White, Andy Muggleton, Mel Morris, Nicholas M. Selby, Maarten W. Taal

**Affiliations:** 1 School of Health and Social CareUniversity of Derby 2939 DerbyDE22 1GBU.K; 2 Centre for Kidney Research and InnovationUniversity of Nottingham 6123 NottinghamNG7 2RDU.K; 3 Renal UnitRoyal Derby Hospital 156672 DerbyDE22 3NEU.K; 4 MStart Foundation and iTrend Medical Research Ltd.DerbyDE24 8DZU.K

**Keywords:** Blood pressure measurement, extracorporeal pressure sensors, fistula, hemodialysis, continuous blood pressure measurement

## Abstract

*Objective:* Intradialytic haemodynamic instability is a significant clinical problem, leading to end-organ ischaemia and contributing to morbidity and mortality in haemodialysis patients. Non-invasive continuous blood pressure monitoring is not currently part of routine practice but may aid detection and prevention of significant falls in blood pressure during dialysis. Brachial blood pressure is currently recorded intermittently during haemodialysis via a sphygmomanometer. Current methods of continuous non-invasive blood pressure monitoring tend to restrict movement, can be sensitive to external disturbances and patient movement, and can be uncomfortable for the wearer. Additionally, poor patient blood circulation can lead to unreliable measurements. In this feasibility study we performed an initial validation of a novel method and associated technology to continuously estimate blood pressure using pressure sensors in the extra-corporeal dialysis circuit, which does not require any direct contact with the person receiving dialysis treatment. *Method:* The paper describes the development of the measurement system and subsequent *in vivo* patient feasibility study with concurrent measurement validation by *Finapres Nova* physiological measurement device. Real-time physiological data is collected over the entire period of (typically 4-hour) dialysis treatment. *Results:* We identify a quasi-linear mathematical function to describe the relationship between arterial line pressure and brachial artery BP, which is confirmed in a patient study. The results from this observational study suggest that it is feasible to derive a continuous measurement of brachial pressure from continuous measurements of arterial and venous line pressures via an empirically based and updated mathematical model. *Conclusion:* The methodology presented requires no interfacing to proprietary dialysis machine systems, no sensors to be attached to the patient directly, and is robust to patient movement during treatment and also to the effects of the cyclical pressure waveforms induced by the hemodialysis peristaltic blood pump. This represents a key enabling factor to the development of a practical continuous blood pressure monitoring device for dialysis patients.

## I. Introduction

Patients receiving hemodialysis treatment as a result of end-stage kidney disease (ESKD) are at a much higher risk of mortality due to cardiovascular disease (CVD) [1]. A key factor contributing to CVD is intradialytic hypotension, a frequent complication affecting 15% – 50% of treatments which is associated with subsequent vascular access thrombosis, inadequate dialysis dose, cardiac dysfunction and mortality. The continuous monitoring of blood pressure (BP) during dialysis, particularly with respect to early detection and prediction of hypotension [2] has the potential to significantly improve patient outcomes [3]–[5] and could ultimately inform the choice of therapeutic intervention via modulation of dialysis time and/or duration, dialysate sodium concentration and/or temperature on a per patient basis.

*Intradialytic* hypotension (IDH) is a sudden event, and generally defined as a decrease in systolic blood pressure greater than $20~mmHg$ or a decrease in mean arterial pressure by $10~mmHg$ [6]. Associated symptoms can include dizziness or fainting, anxiety, muscle cramps, abdominal discomfort, nausea and vomiting. In addition to the negative impact on patient well-being, IDH can result in truncated dialysis treatments and increase the risk for coronary and cerebral ischemic incidents. IDH prediction, detection and mitigation is ultimately the primary application area of the work described in this article. Continuous monitoring of blood pressure (BP) during dialysis could ultimately inform the formulation of a treatment regime, or close a personalised therapeutic intervention loop via modulation of dialysis time and/or duration, dialysate sodium concentration and/or temperature on a per patient basis.

Arterial cannulation is widely regarded as the *gold standard* reference for continuous measurement of BP [7], (which is referenced to brachial artery BP in common with all the methods discussed here). While a common procedure during high-risk surgery, it is not considered appropriate for haemodialysis patients where non-invasive monitoring is indicated. This is normally achieved in a clinical setting via the use of an air-filled occluding arm cuff that provides a robust, but intermittent measurement that disrupts the normal blood flow, and so requires a significant settling time between measurements [8].

Three distinct methods for non-invasive monitoring of BP have occasionally been used in research settings. The first of these is arterial applanation tonometry [9] where a transducer is positioned above a superficial artery compressing it against an underlying bone. Analysis of the resulting pulse wave has been extended to calculate systolic and diastolic pressure [10]. This method has been used in cardiology and anaesthetised procedures to avoid the insertion of an arterial cannula [11], [12], but as these devices are hand-held and are operator-dependent, they are unsuitable for continuous monitoring.

The second method for non-invasive continuous estimation of BP is Pulse Transition Time (PTT) [13] which is based on measured photoplestimography (PPG) and electrocardiogram (ECG) signals during several cardiac cycles. PTT is then calculated as the time difference between the ‘R’ peak in the ECG signal and the corresponding time instance of the inflection point on the maximum slope of the PPG signal. This method may not be accurate due in part to the unaccounted physiological factors in the blood regulation mechanism and reliance on accurate ECG triggering [14].

The third alternative is the volume clamp (or vascular unloading) method [8] where an inflatable finger-cuff combined with an embedded photodiode measures the diameter of the finger artery. Cuff pressure is adjusted to maintain a constant artery diameter, and the changes in cuff pressure are used to calculate a BP curve in the brachial artery. In use, patients frequently report pain or discomfort at the fingertips where cuffs are placed, and this device can be unreliable in patients with reduced blood flow to the digits, for example, dialysis patients [13]. All three non-invasive methods are sensitive to patient movement (especially PPG based methods) [15] which would result in an unacceptable and uncomfortable restriction placed on patients during a four-hour dialysis treatment.

### A. The iTrend Renal Dialysis Programme

The iTrend (Intelligent Technologies for Renal Dialysis) programme is a long-term collaborative project performed by a multidisciplinary research team from the Universities of Derby and Nottingham and the Royal Derby Hospital Renal Unit in the UK. The primary goal of the programme is to develop supporting technologies and real-time analysis of data to enable personalised and precision treatment in ESKD [16], [17]. Blood pressure monitoring to diagnose hypertension and hypotension [18], [19] are typically plethorperidialytic and intermittent intradialytic BP measurements. The ability to continuously monitor BP both in terms of absolute measures and blood pressure variability could lead to new diagnostic and prognostic criteria for IDH as well as optimal and personalised treatment strategies.

In this work, a feasibility study based around a novel method for continuously monitoring BP is described which involves the placement of pressure transducers onto the venous and arterial blood lines that connect a patient to a haemodialysis machine during treatment. This study is the first step to develop a continuous blood pressure estimating/measuring device for dialysis treatment. The resulting data is compared against standard BP measurements obtained via an occluding arm cuff and the volume clamp method, via the Finapres Nova (FMS, Finapres Measurement Systems, Arnhem, Netherlands), whose accuracy has been studied [9], [10] and validated according to the recent British Hypertension Society protocol and the criteria of the Association for the Advancement of Medical Instrumentation. Only the intermittent cuff pressured recorded by Finapres Nova is utilised in the present manuscript

### B. Structure of This Article

This article describes the development of a continuous, non-invasive method to measure BP during renal dialysis sessions. Design objectives were as follows:

• to provide reliable and robust measurement of patient blood pressure before, during and immediately after haemodialysis treatment;
• with regards to patient experience, to require no extraneous sensors to be attached directly to the patient;
• the measurement method to be robust to patient movement during treatment;
• the sensors to be cost-effective, non-bespoke and readily available as off the shelf items;
• data aquisition and analysis hardware to be low physical footprint and low cost;
• the system should not require any special interfaces to and should work equally well with any proprietary dialysis machine.

The paper is divided into the following sections:

• Section II: A generalised parameterised model is derived to describe the approximate relationship between measured brachial cuff pressure measurements and arterial line pressure measurements during hemodialysis treatment.
• Section III: The feasibility study data acquisition system and associated hardware is described along with experimental study participants and the dialysis bay experimental setup.
• Section IV: Results from the experimental feasibility study are graphically displayed and analysed.
• Section V: Conclusions derived from the study are presented, and future work described.

For clarity, where a patient’s BP is mentioned, this should be taken to mean brachial blood pressure.

## II. Continuous, Non-Invasive Intradialytic BP Measurement From Dialysis Lines

The use of pressure signals from the arterial and venous blood lines of a dialysis machine has been shown in principle to enable continuous online monitoring of a patient’s heart rate, even for patients with low cardiac signal amplitude [20], [21]. The method has been extended to monitor ventricular premature beats [22]. Performance was studied for one dialysis flow rate ($400~ml/min$), and results lacked fidelity where patient heart rate and haemodialysis pump speed coincide. While promising, the method generated to a number of false positive and false negative predictions of IDH.

Our approach demonstrates a more robust approach to derive continuous BP signals from the arterial and venous dialysis lines and presents the initial results of its in-vivo verification via on-going patient studies in the Renal Unit at Royal Derby hospital.

### A. Method

The aim of this feasibility study is to identify and experimentally derive relationships between the patient’s brachial BP, and the pressures recorded within the extra-corporeal blood lines to and from the dialysis machine. Typically, two such blood lines are provided during haemodialysis: an arterial line conducts blood from the patient to the dialysis machine, and a venous line that conducts blood from the dialysis machine back to the patient. Vascular access to the patient is provided by means of two large gauge needles (one on each line) into the patients ‘fistula’, a surgically enlarged blood vessel resulting from the connection of a vein onto an artery, typically located within the patients non-dominant arm.

**FIGURE 1.**
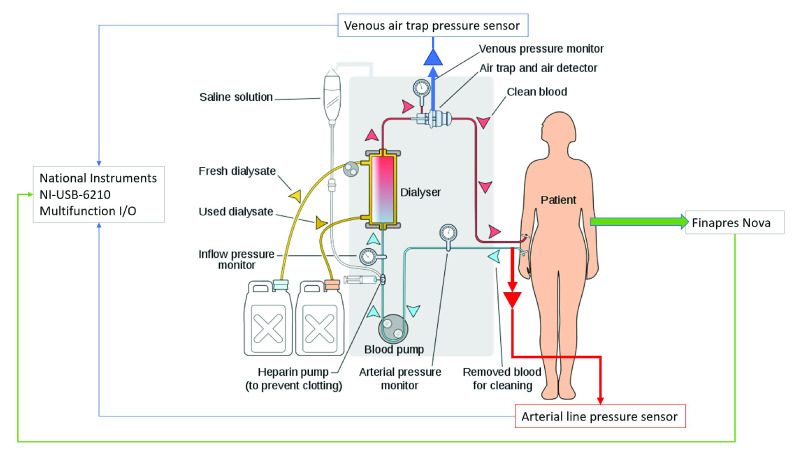
Sensor and data acquisition signal flow.

In order to develop a non-hardware specific generalised solution to the problem, additional pressure sensors were placed into the blood lines rather than making use of the pressure sensors present within the dialysis machines (as in previous studies). The primary function of these safety critical machine sensors is setup monitoring. This study utilised the existing ports and connection points which are common and standardised on most dialysis blood lines sets (Fig 1).

**FIGURE 2.**
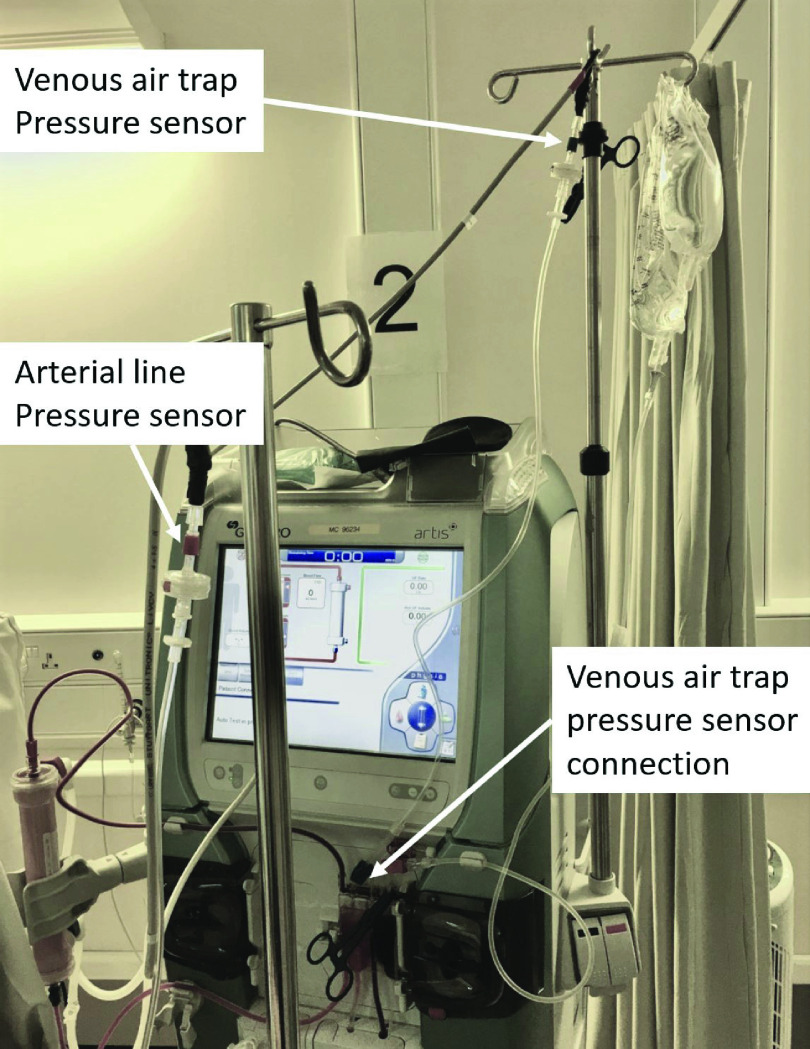
Pressure sensor connection to venous air trap port.

Starting on the blood return side (Fig. 1) venous blood line provides a standard 4 mm line connection point on the air trap, which was chosen as the site for the additional venous line pressure sensor. This is typically the final port on the venous side (Fig. 2).

As will be seen, the blood flow rate through the extra-corporeal system is critical to analysis of the relationship between brachial and arterial line blood pressure measurements. A real-time measurement of blood flow is required for the calculations necessary for BP estimation. Flow rate is generally set at the beginning of treatment, and recorded manually. For various clinical reasons, medical professionals may adjust the flow rate during treatment which may or may not be recorded in patient notes.

The pressure waveform measured by the pressure sensor connected to the venous bubble trap is dominated by the oscillations introduced by the lobes of the peristaltic blood pump [23]. The periodic pressure waveform of such pumps results from alternative compression and relaxation of the dialysis line (typically $8~mm$ diameter at the pump) by the (typically 2) lobes of the pump. Hence the pump frequency can be derived from a real-time positive pressure sensor sited in the venous air trap by the application of Fourier analysis. By this method, any reasonably well-behaved function can be written in terms of trigonometric or exponential functions.

Considering a function $f(x)$ that is periodic on the interval $0 \leq x \leq L$, Fourier’s theorem states that $f(x)$ can be written as the *Fourier trigonometric series* for the function as;

\begin{equation*} f(x)=a_{0} \sum _{n=1}^{\infty } \left [{a_{n}\cos \left ({\frac {2 \pi n x}{L} }\right) + b_{n}\sin \left ({\frac {2 \pi n x}{L} }\right) }\right]\tag{1}\end{equation*}

As $2\pi $ is included in the arguments of the trigonometric functions, then the $n = 1$ terms have period $L$, the $n = 2$ terms have period $L/2$, etc. for higher harmonics. For any integer $n$, an integral number of oscillations fit into the period $L$. In the case under examination here, the fundamental frequency of the blood pump is sought, so calculations are conducted for $n=1$.

The pump frequency is calculated and updated in real-time over a sliding window of data which is *5000 samples* or *5 s* wide. As the pump frequency is typically around $1~Hz$, this ensures sufficient data, without including dynamics, so effectively a *quasi steady-state* measurement. Frequency is converted to flow in units of microlitres/minute by

\begin{equation*} fl=\left ({\left ({\frac {fr(rads^{-1})}{2\pi }}\right)\times 60 }\right) \times \left ({\pi r^{2}Ln}\right)\tag{2}\end{equation*}

where $fl$ is flow in $mls^{-1}$, $fr$ is pump frequency in $rads^{-1}$, $r$ is the radius of the dialysis line within the pump in $mm$ and $Ln$ is the effective length of line within the pump in $mm$. Brachial pressure is regularly measured via inflatable cuff, and data for all the sensors is synchronised and stored via a National Instruments data acquisition device.

**FIGURE 3.**
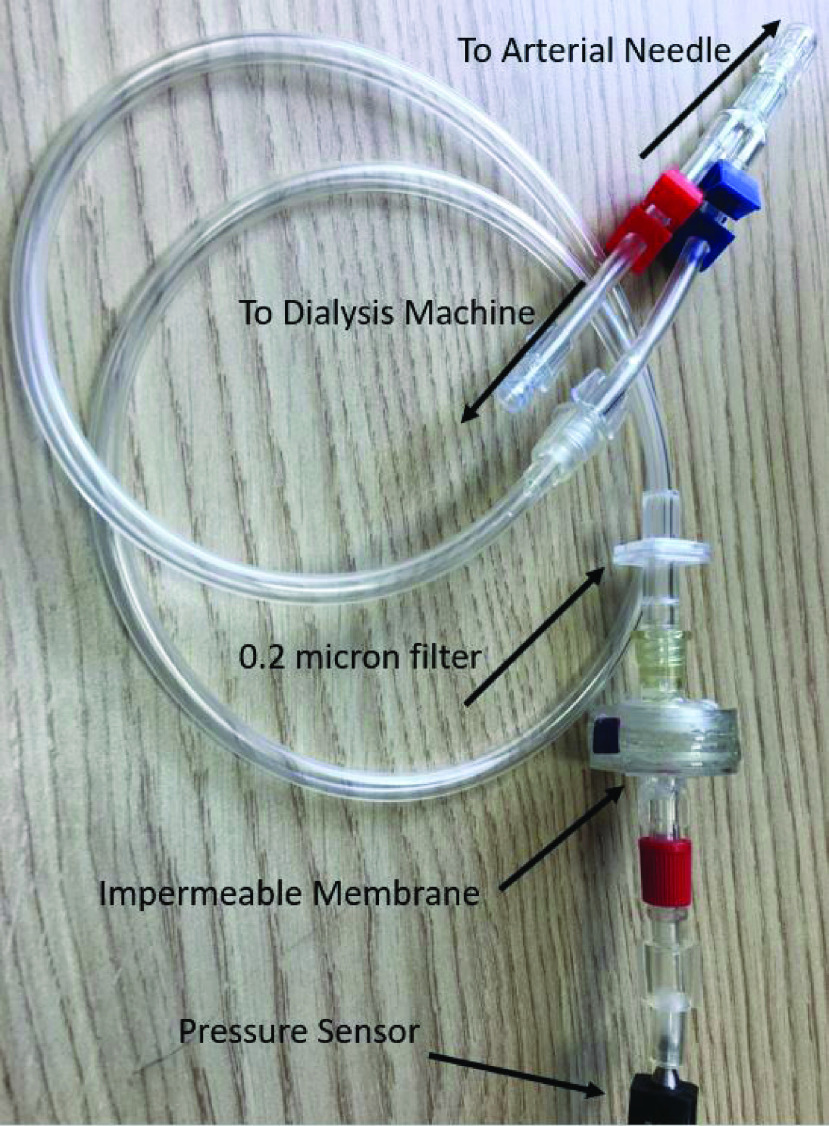
Arterial line pressure sensor connector.

The additional pressure sensor on the arterial-line should be sited as close as possible to the patient’s vascular access, since the needle sits between positive pulsatile patient blood pressure at one end, and blood-pump dominated negative pulsatile pressure at the other end. Given that no suitable connector exists at the arterial needle end of the line, a ‘Y’ connector with 4 mm internal diameter is fitted to the tubing of the dialysis needle to allow access to both the dialysis line and the arterial line pressure sensor (Fig. 3).

**FIGURE 4.**
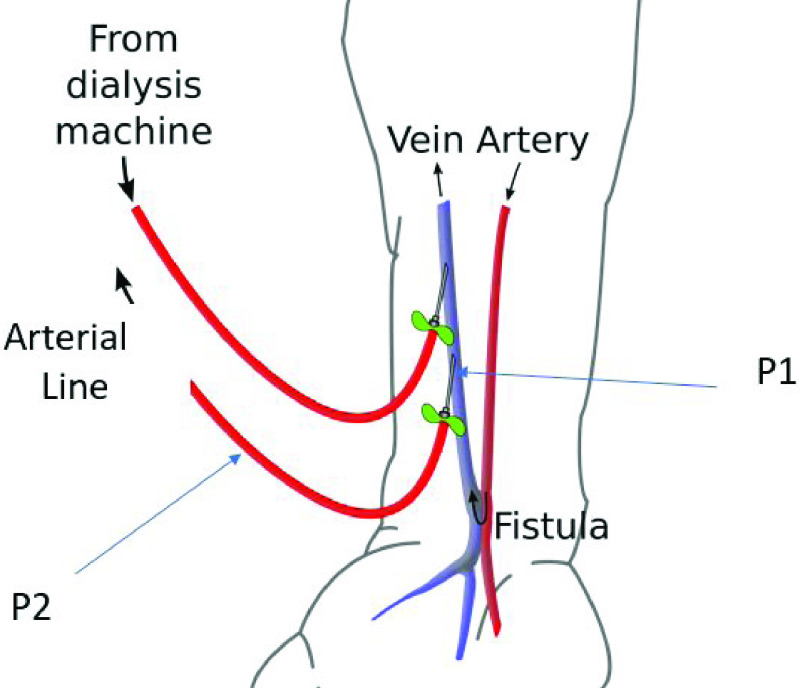
Arterial line and needle pressure datum points.

Modelling the relationship between arterial-line pressure and brachial pressure is extremely complex with high levels of physiological differences between patients. In order to produce a tractable model, a number of approximations are included. The brachial cuff measurement is, in effect, a quasi-steady state measurement single-instance sample of systolic and diastolic pressure including no dynamic information, and hence the arterial line pressure is filtered by *5000 samples moving-average* window, similar to the filter applied to the venous-line pressure data. With reference to Fig 4, datum point 1 is defined as the tip of the arterial line needle, and associate pressure $P_{1}$ and area $A_{1}$ with it. Similarly, datum point 2 is defined at the pressure sensor, and associate pressure $P_{2}$ and area $A_{2}$ with it.

Assuming steady, incompressible flow with negligible losses, blood flow rate can be expressed using Bernoulli and Continuity equations [26] as;

\begin{equation*} f=A_{2}\left [{\frac {2\left ({P_{1}-P_{2}}\right)}{R\left ({1-\left ({\frac {A_{2}}{A_{1}}}\right)^{2}}\right)}}\right]^{\frac {1}{2}} \tag{3}\end{equation*}

where f is fluid flow and R is the fluid density.

A number of assumptions are inherent in this model; horizontal flow and fully developed flow at $P_{1}$ and $P_{2}$, density and viscosity are constant wrt time. However, many, if not all, of these assumptions are either unmeasurable, or vary in practice from patient to patient, or are time-varying during treatment. Additionally, the temporal and physical distance between the fistula needle site and the measurement site at the brachial cuff (typically on the opposite arm to the fistula) is significant ($\tilde 1~m$). Even for well defined problems, theoretical flow $f$ is generally 2% – 40% lower than empirical due to geometry. Therefore, (3) is modified as

\begin{equation*} f=A_{2}\left [{\frac {2\left ({P_{1}-P_{2}}\right)}{C}}\right]^{\frac {1}{2}} \tag{4}\end{equation*}

where $C$ is an experimentally determined lumped-parameter which approximates the unknown and unmodelled features of the system and the unmet assumptions of the analysis. The lumped parameter inherently includes the effect of different dialysis procedural details such as the needle gauge size that may be used with different patients, and blood pump flow-rate according to different treatment prescriptions. Individual patient-specific parameters such as blood viscosity, density and temperature are also implicit. Rearranging for $C$

\begin{equation*} C=\frac {2\left ({P_{1}-P_{2}}\right)}{\left ({\frac {f}{A_{2}}}\right)^{2}} \tag{5}\end{equation*}

the expression allows computation of $C$ from measured values. Assuming $P_{2}$ represents measured arterial line pressure as before, and the lumped unknown parameters coefficient $C$ includes the unknown relationship between $P_{1}$ and measured brachial pressure $P_{b}$, then a quasi-linear relationship between arterial line pressure and brachial pressure can be derived from equation (5), if and only if the unknowns represented by the variable $C$ are accurately modelled and time-invariant. The relationship is given by

\begin{equation*} -P_{2}=0.5\left ({\frac {f}{A_{2}}}\right)^{2}C-P_{b}\tag{6}\end{equation*}

where $C$ defines the gradient of the relationship and can subsequently be used as a predictor between measured $P_{2}$ and estimated $P_{b}$. Given the expectation that C will contain time varying and unmodelled terms, it can be predicted that there will be variation around the mean for individual patient measurements. Future and continuing work may identify a more accurate description of the system, however the linear model derived here and presented in the study data confirms the feasibility of the approach.

## III. Experimental Hardware

Low-cost industrial process control pressure sensors with on-board signal amplification and linearization (Honeywell *40PC015V2A* 0 PSI to −15 PSI Arterial line, and Honeywell *40PC015G1A* 0 PSI to 15 PSI Venous line) have been integrated (Fig 1 ^1^) with connectors to fit ports on standard dialysis lines. The connectors consist of a $4~mm$ internal diameter line, a pressure transducer protector which keeps the blood side of the circuit separated from the sensor, preventing patient cross contamination with blood borne pathogens via a *0.2 micron* filter. There is also a bespoke membrane barrier (Fig. 3) fitted as an extra safety precaution. [24], [25]^1^after: https://commons.wikimedia.org/wiki/File:Hemodialysis-en.svg

Real-time data acquisition (DAQ) and storage is performed via a National Instruments *NI USB-6210*,^2^ 16 input 16-bit, $250~kS/s$ multifunction I/O device. All acquisition is analogue, sampled at $1~kHz$ on each line. Analogue input lines operate in the range 0 to $5~V$. Data from the Finapres Nova is transferred to the National Instruments device via dedicated multicore cable. The following Finapres Nova analogue output lines were acquired by the DAQ hardware:

• Reconstructed systolic pressure
• Reconstructed diastolic pressure
• Finger arterial pressure
• ECG II waveform
• Reconstituted brachial arterial pressure
• Brachial arm-cuff measurements

Additionally the venous air trap pressure sensor and arterial line pressure sensor are connected via dedicated shielded cables, which include $+5\,\,V$ and $0~V$ supplies to power the sensors from the acquisition device. All data streams are time synchronised via a common real-time clock.^2^https://www.ni.com/pdf/manuals/375194d.pdf

### A. Experimental Study

Method: Participants > 18 years were recruited from the prevalent dialysis population at Derby Royal Hospital. Patient’s demographics, dialysis background, dialysis prescription, laboratory parameters pre- and post-dialysis at each session were recorded. All participants had continuous non-invasive monitoring of haemodynamics using pulse wave analysis (Finapres NOVA) during a single dialysis treatment of typically 4 hours duration, and this data was periodically verified by intermittent arm-cuff measurements. Two pressure sensors were attached to the dialysis lines as described: one between the arterial dialysis needle and dialysis line and the other directly onto the venous-line bubble trap. Data was continuously recorded from all sensors at a sampling rate of 1 kHz.

The study protocol was approved by the West Midlands - Coventry and Warwickshire Research Ethics Committee and participants gave written informed consent.

## IV. Results

In total 12 participants were recruited with 11 completed monitored dialysis sessions. 1 participant moved out of area before a session could be completed. 58.3% of participants were male, with a median age of 65 (IQR 48-78).

**FIGURE 5.**
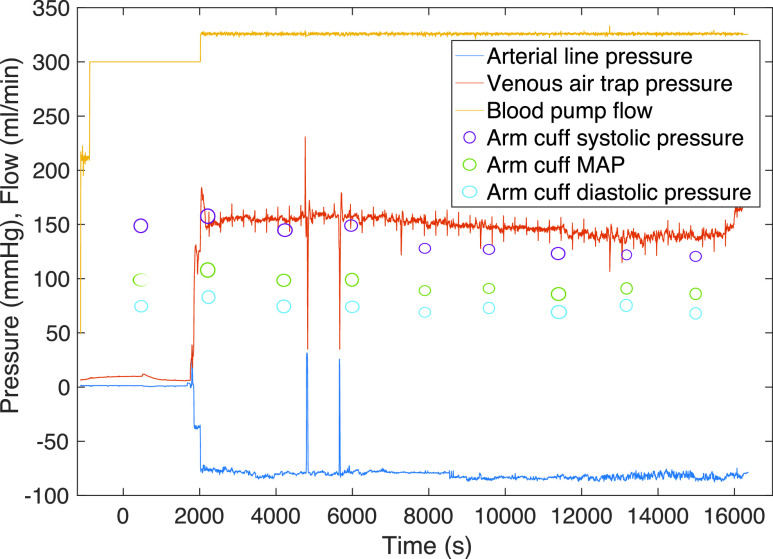
Typical data output for 4-hour treatment session.

Serial brachial cuff blood pressure values were compared with synchronous pressure sensor data in Matlab (ver. 19b). Arterial line pressure sensor data was analysed for dialysis pump flow by fitting a Fourier model to the continuous venous line pressure signal. Derived pump flow, arterial and venous line pressures, and brachial cuff pressure measurements for a typical treatment session are shown in Fig. 5. Values from the brachial arm cuff (*Systolic, Diastolic and MAP*) are saved to a master record with synchronised time stamps for comparison with the corresponding venous and arterial line pressures. This master record for the patient study enables the calculation of lumped parameter $C$.

In this example (Fig. 5), the dialysis blood pump is enabled at $2000~s$ resulting in divergence of the arterial ($-ve$) and venous ($+ve$) line pressures. Subsequently, the Fourier model outputs a flow rate of $325~ml/min$ which remains constant for the duration of the treatment, and agrees with the patient record. Brachial cuff BP measurements are taken from approximately 30 minutes before the start of treatment and repeated approximately every 30 minutes throughout. The cuff measurements of systolic and diastolic BP and MAP are recorded via the DAQ system with a corresponding value for blood flow and for arterial pressure. In this case, it is possible to construct a table of 8 measurements for this treatment, and a master data set can thus be constructed for the 11 completed dialysis sessions which allows an analysis to be conducted against a varied participant population.

In the Renal Unit at Royal Derby Hospital, where the patient studies are conducted, 14g is the standard and preferred gauge size for dialysis patients. Smaller diameter (larger gauge size) needles are used for new patients with recent fistula, and also for patients with needling problems.The gauge sizes used during this patient study are:

• 14: 14g Arterial 14g Venous
• 15: 15g Arterial 15g Venous
• 16: 15g Arterial 16g Venous

where 14 is the normal gauge set, and 15, 16 used for new patients and access issues.

**FIGURE 6.**
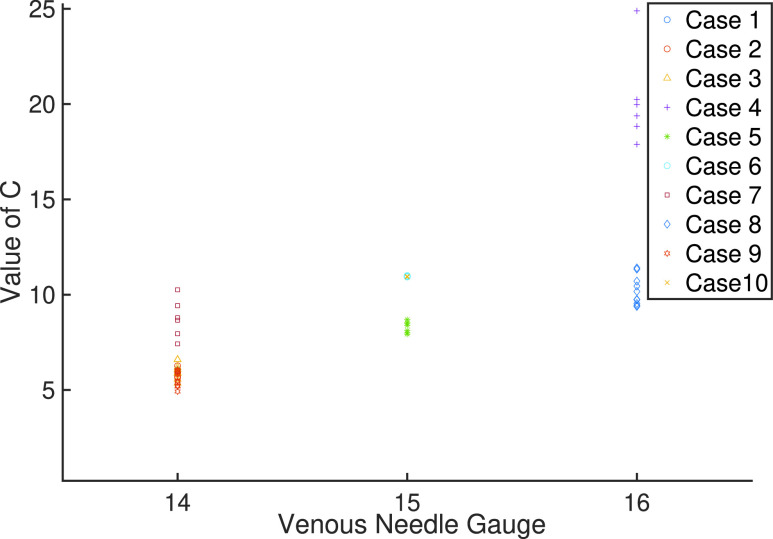
Values of $C$ associated with Arterial and Venous needle sizes.

With reference to Fig. 6, $C$ values are calculated for every measurement set in each treatment, and grouped according to patient’s needle gauge sizes, which shows the fundamental effect of needle gauge size on the relationship between $P_{b}$ and $P_{2}$ measurements. Fig. 6 also illustrates a number of salient features. In particular, a relatively tight core grouping of $C$ values for each needle size set (14: 5 – 7, 15: 8 – 9, 16: 9.5 – 11). A rising mean of this core grouping (6, 8.5, 10.25) indicates the influence of the reducing available area on the difference between $P_{b}$ and $P_{2}$. As expected, Fig. 6 shows a number of outlying measurements. In particular for needle gauge size 16, the outliers are all from a patient with a relatively new arteriovenous fistulae (AVF), and may indicate that maturation of the blood vessel is not complete [27]. For all gauges, it is salient that the coefficient $C$ describes a range of linear and nonlinear unmodelled effects beyond needle gauge size and would be expected to contain outliers in a physiologically non-homogenous patient study population and can additionally contain time varying effects experienced over the duration of a 4 hour treatment session. With respect to venous needle gauges in the study, there were 5 cases with 14g, 3 cases with 15g and 2 cases with 16g. The relationship to, and deviation from the mean value of C wrt needle gauge is of interest and will be investigated in further studies.

**FIGURE 7.**
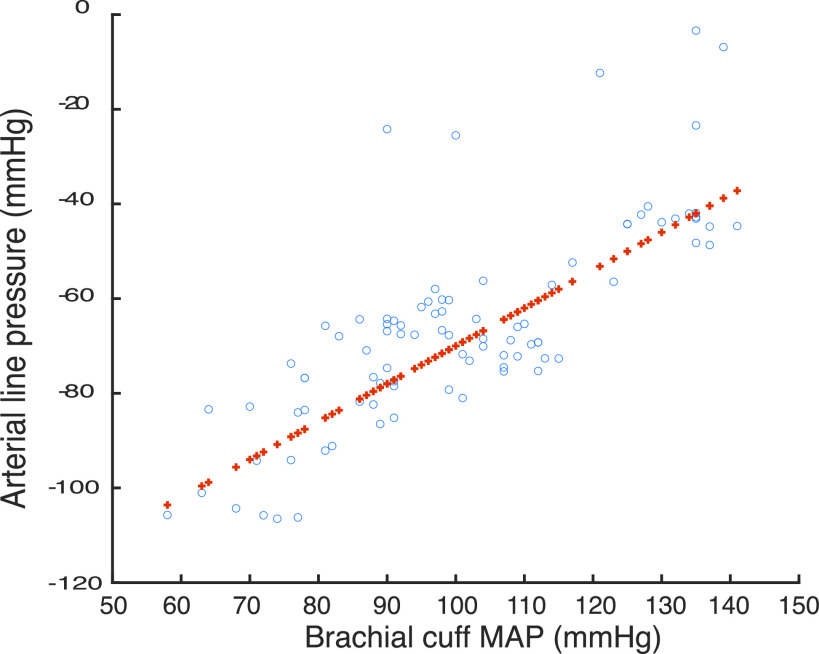
Brachial cuff MAP vs Arterial line pressures in 11 patient sessions (o) with least squares linear fit (+).

The experimental relationship between brachial cuff MAP and corresponding measured arterial line pressure for 11 sessions in the patient study is shown in Fig. 7. A linear least squares fit of $y=0.8x-1.5*10^{2}$, Root Mean Squares Error 10.3, $R^{2}~0.615$, p-value 6.35 * 10^−20^. As predicted, the figure displays a correlated scatter for the population around a linear least squares fit with a gradient which represents a fundamental relationship between $P_{b}$ and $P_{2}$. This is common to all members of the study, and hence is strongly associated with the physical components of the dialysis procedure (pump, pump speed, dialysis line set), with variance from the line dominated by unmodelled physiological effects. This is a significant outcome for the project, as it means that the process of real-time prediction can be decomposed into two separate functions. Namely, updating the machine mean relationship to take account of any hardware variances between machine sets (new machine and or lines can be modelled into a library), and learning the relationship to the machine line on a *per patient* basis (which again can be an updateable model).

**FIGURE 8.**
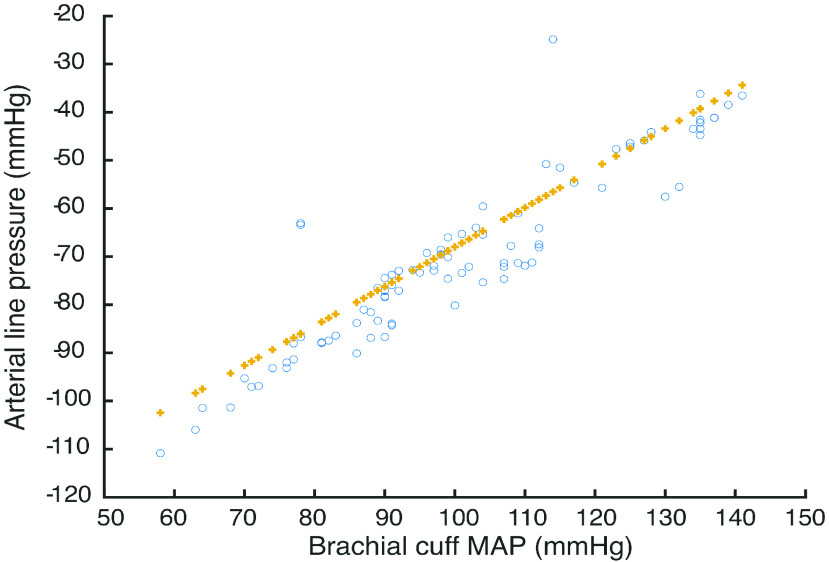
Brachial cuff MAP vs Arterial line pressures in 11 patient sessions (o) with least squares linear fit (+) following compensation for time varying effects during treatment.

In order to further examine this hypothesis, it is possible to negate the time varying effects in the data by taking a mean value for $C$ for each individual patient treatment, and recalculating the data scatter. This should reduce data variance since the scatter around the least squares fit line consists of two components: time varying effects, and unmodelled physiological effects, and also to a certain extent negate the time varying effects in the data by taking a mean value for $C$ for each individual patient treatment, and recalculate the data scatter. This is shown in Fig. 8. A linear least squares fit of $y=0.82x-1.5*10^{2}$, Root Mean Squares Error 6.54, $R^{2}~0.852$, p-value 2.93 * 10^−38^. There are a number of salient features in this plot. Firstly, the gradient of the fit has only changed by 2.5% indicating a relatively accurate model for the machine and lines system. Secondly, the data $R^{2}$ value has risen by 38.5% to 0.852, indicating that a tractable and useable time-invariant model can be constructed. The flow estimator calculations corresponded with dialysis machine settings within the band +/−1%. The patients under observation within this study all received constant flow rate throughout individual treatments (cf Fig. 5). However, the range of flow rates across the patients in the study was $278-394\,\,ml/min$, which was identified by the estimator in each case contributing to the level of accuracy shown in Fig. 8. Flow changes during treatment will be built into the next planned patient study.

Finally, although the majority of physical systems can be modelled adequately by linear, time-invariant models, there is much to be gained in future work developing time-variant, non-linear models of these specific systems. Overall, data from this first feasibility study indicates that a linearised model for individual patients, patient populations and machine sets can be calculated which defines a linear relationship between measured arterial line pressure and measured brachial artery pressure. The feasibility study thus confirms that it is practically possible to estimate brachial BP continuously and non-invasively during the period of a dialysis session in a clinical setting with measurement of venous and arterial line pressure, and occasional measurement of brachial blood pressure by arm cuff.

## V. Conclusion

The objective of this patient study was to answer a pressing question in dialysis treatment, whether a method could be developed which demonstrates the feasibility of continuous non-invasive blood pressure monitoring, without compromising patient experience and without requiring bespoke interfacing to the dialysis machine, or any additional sensing to be worn by the patient beyond current arm cuff monitoring requirements.

An approximate mathematical model relating arterial line pressure, blood pump flow rate and brachial pressure was derived, which predicts a quasi-linear relationship between arm cuff measured pressure, and corresponding pressure measured in the arterial line near to the fistula. Interfaces, measurement devices and a data acquisition were designed and built to support an observational study during renal dialysis treatment.

This is an early study, and we felt that a formal analysis of external disturbances would form part of a later study. At this time however, we can state that during each of the cases studied here, our method provided a continuous data record over the treatment period, whereas the data from the Finapres Nova was restricted to 30 minute sessions with 30 minute breaks due to discomfort. Additionally the Finapres Nova data exhibited many instances of waveform discontinuities and settling-time/re-calibration in the data sets.

The results from this observational study suggest that it is feasible to derive a continuous measurement of brachial pressure from continuous measurements of arterial and venous line pressures via an empirically based and updated mathematical model. Potential exists to identify a more complex and hence more accurate parametric model.

The study presented here is critical to confirming that the fundamentals of this approach are feasible, and the results have confirmed that the methodology and technology has a practical future in renal units. There are a number of challenges following directly from this work which will be addressed in current and future algorithmic/technological developments and studies. In particular, iterative learning algorithms to update the mathematical models based upon incoming cuff BP measurements, improved mathematical models to increase estimation accuracy, and predictive models for hypotension. Patient studies are about to start at Royal Derby Hospital to demonstrate, analyse and improve the continuous estimation of brachial BP in *real-time* for the complete duration of dialysis treatments. Further work will report on the outcome of these studies.
